# Combining Time-Driven Activity-Based Costing with Clinical Outcome in Cost-Effectiveness Analysis to Measure Value in Treatment of Depression

**DOI:** 10.1371/journal.pone.0165389

**Published:** 2016-10-31

**Authors:** Samir El Alaoui, Nils Lindefors

**Affiliations:** Department of Clinical Neuroscience, Centre for Psychiatric Research, Karolinska Institutet, Stockholm, Sweden; University of Illinois at Chicago College of Medicine, UNITED STATES

## Abstract

**Background:**

A major challenge of mental health care is to provide safe and effective treatment with limited resources. The main purpose of this study was to examine a value-based approach in clinical psychiatry when evaluating a process improvement initiative. This was accomplished by using the relatively new time driven activity based costing (TDABC) method within the more widely adopted cost-effectiveness analysis framework for economic evaluation of healthcare technologies. The objective was to evaluate the cost-effectiveness of allowing psychologists to perform post-treatment assessment previously performed by psychiatrists at an outpatient clinic treating depression using internet-based cognitive-behavioral therapy (ICBT).

**Methods:**

Data was collected from 568 adult patients treated with ICBT for depression during 2013–2014. The TDABC methodology was used to estimate total healthcare costs, including development of process maps for the complete cycle of care and estimation of resource use and minute costs of staff, hospital space and materials based on their relative proportions used. Clinical outcomes were measured using the Patient Health Questionnaire depression scale (PHQ-9) before and after treatment and at 6-month follow-up. Cost-effectiveness analyses (CEA) was performed and the results presented as incremental net benefits (INB), cost-effectiveness acceptability curves (CEACs) and confidence ellipses to demonstrate uncertainty around the value of the organizational intervention.

**Outcomes:**

Taking into account the complete healthcare process (from referral to follow-up assessment), treatment costs decreased from $709 (*SD* = $130) per patient in 2013 to $659 (*SD* = $134) in 2014 while treatment effectiveness was maintained; 27% had achieved full remission from depression after treatment (PHQ-9 < 5) during both 2013 and 2014 and an additional 35% and 33% had achieved partial remission in 2013 and 2014, respectively. At follow-up, 42% were in full remission after treatment during both 2013 and 2014; an additional 35% and 33% were in partial remission during 2013 and 2014, respectively. Confidence ellipses occupied the south-east (SE) and south-west (SW) quadrants of the incremental cost-effectiveness plane at both post-treatment and at follow-up, indicating that the ICBT treatment was less costly and equally effective after staff reallocation.

**Conclusion:**

Treating patients to the target of full remission using psychologists instead of medical specialists for post-treatment assessment is cost-saving and consequently a more valuable use of limited resources. TDABC may be a useful tool for measuring resource costs, identifying quality improvement opportunities and evaluating the consequences of such initiatives. Combining TDABC with clinical outcome measures in CEA is potentially a useful approach in mental healthcare to estimate the value of process improvement initiatives.

## Introduction

The social and economic effects of the growing global burden of mental disorders—notably, depression—are monumental. The 2010 Global Burden of Disease (GBD) study identified depression as the second-leading cause of disability worldwide, concluding that depressive disorders are a public-health priority requiring cost-effective interventions.[1] To meet the increasing demands on health care services, care delivery must be organized to maximize resource efficiency without compromising effectiveness and patient safety. An innovative solution, with the potential to bridge the gap between demand and accessibility of qualified treatment for mental health problems, is the recent development of internet-based psychotherapies, which use established evidence-based treatment programs, such as cognitive behavioral therapy (CBT)[2], in combination with online therapist support. Such interventions are generally referred to as internet-based CBT (ICBT). [3, 4] Treatment components are similar to conventional face-to-face CBT (e.g., educating patients about depression and rationale for the CBT model of treatment, goal setting, problem solving, behavioral activation, cognitive restructuring and relapse prevention), with the difference being the novel use of internet-based communication between patient and therapist. Because ICBT is less resource demanding than conventional face-to-face interventions—and does not compromise treatment effectiveness—it could greatly increase access to evidence-based and cost-effective care, complementing traditional forms of delivery.[5]

With an increased emphasis on mental health care in both assuring the quality of care while also managing costs, there is a corresponding need for a management system that can accurately and routinely estimate these factors. Access to detailed and reliable information on both costs accrued and treatment results achieved is critical for healthcare providers seeking to implement a value-based approach in which these critical dimensions are continuously monitored.[6, 7] Whereas measuring clinical outcomes is relatively straightforward, measuring resource use and the total costs of treating patients is more complex. Therefore, appropriate costing methods, which take the inherent complexities of health care systems into account, should be used in such estimations. Earlier studies on costing in healthcare have mainly focused on traditional hospital settings.[8–12] As such, there has been little research on cost systems in primary care and, specifically, in psychiatry. A recently developed method that may increase the accuracy of treatment cost measurement is time-driven activity-based costing (TDABC).[13] Advantages of TDABC may include easier identification of inefficiencies and clinical improvement opportunities since a detailed inventory of each step of the care process is documented and evaluated.[7] However, for a process improvement initiative to be of *value*, the impact of increased efficiency and lowered healthcare costs on the quality of care and on the clinical effectiveness of the healthcare process should always be evaluated.

To visualize the value of different treatment strategies or healthcare options, the cost-effectiveness plane (CE plane) has been widely used as a standard tool where differences in both costs and clinical outcomes between two options are illustrated [14]. The originators of the value-based healthcare framework propose that organizations use radar charts to illustrate value, since such graphs can communicates changes in costs and outcomes on several different dimensions simultaneously [15]. However, to be able to determine the accuracy or uncertainty surrounding the value created through process improvement initiatives, cost-saving strategies or quality improvement initiatives, we believe that the more widely adopted and recommended methods for cost-effectiveness analysis (CEA) [16, 17] coupled with the CE plane with confidence ellipses [18] should be used in any attempt to quantify and interpret the meaning of “value” in value based healthcare. Consequently, integrating TDABC into CEA, thus measuring value in accordance with recommended methodological guidelines, has been the focus of this study. The object of study was the evaluation of a process improvement initiative at an outpatient psychiatric unit treating anxiety disorders and depression using ICBT.

At the ICBT unit of Stockholm Health Care Services, Psychiatry Division, the effectiveness of ICBT has been well documented for a variety of mental health disorders, including depression.[19–21] The overall quality of care encompasses more than clinical outcomes; dimensions such as treatment accessibility, resource utilization and sustainability of health are equally important. Demand for treatment may vary significantly from year to year where periods with peaking demand typically lead to increased backlogs of undiagnosed patients, consequently increasing waiting times. For example, the waiting list increased by 44% as the number of yearly referrals increased from 1,026 in 2010 to 1,473 in 2013. As a consequence, the unit struggled to meet its internal quality standards of providing patients with a diagnostic assessment within three weeks and treatment availability was therefore lowered. As a process improvement initiative, a reallocation of staff was initiated in late 2013, whereby psychiatrists, who typically managed both pre- and post-treatment assessments, would focus only on clinical visits before treatment, whereas psychologists would conduct the post-treatment assessments. The objective was to increase treatment availability by reorganizing work activities performed by medical doctors allowing them to focus on more acute tasks (i.e. utilizing more of their available clinical time to complete pre-treatment diagnostic assessments) with sustained treatment effectiveness. By allowing a resource with a lower cost rate (i.e. psychologists) to perform a key activity in the healthcare process, one hypothesis was that this staff reallocation would also lead to a cost reduction.

The purpose of this study was to evaluate the usefulness of TDABC as a costing method within the cost-effectiveness analysis (CEA) framework to estimate the value of process improvement initiative for a healthcare provider perspective.

## Methods

### Setting and study design

This study was conducted at a public ICBT unit in Stockholm, Sweden (Stockholm Health Care Services, Psychiatry Southwest). Because of its highly standardized work processes and the high-volume nature of ICBT, an ICBT unit was considered well suited for TDABC and process management modifications. Data from 2013 and 2014 were used to compare outcomes and costs before and after staff reallocation. The research was approved by the Regional Ethical Review Board in Stockholm, Sweden (no 2011/2091-31/3).

### Participants

Data was collected from patients (N = 568) who were treated for depression at the ICBT unit during 2013–2014 and completed post-treatment assessment. A majority of patients were self-referred to the clinic through an online registration and screening system, after which they were invited to a structured diagnostic interview with a clinician. To be included, patients had to (a) fulfill DSM–IV criteria of depression, (b) agree not to undergo concurrent psychological treatments, (c) have a stabilized dose of psychotropic medication for 4 weeks if on medication, (d) be able to read and write, (e) be ≥ 18 years, (f) not present with too complex co-morbid difficulties that might make ICBT an unsuitable intervention (e.g., ongoing substance abuse or a psychotic syndrome), and (g) have access to a computer or other device with an Internet connection.

### Cost measurement

To determine the cost of ICBT care in patients with depression, we followed the methodology of time-driven activity-based costing (TDABC). The following steps were applied in developing the TDABC models for each time period.

#### Step 1: Selecting the medical condition

Patients diagnosed with and treated for depression was selected for this study. The care cycle included follow-up assessment at 6 months to include a measure of the sustainability of achieved treatment effectiveness.

#### Step 2: Defining the care delivery value chain

An overall view of the care process was defined, including the following principal activities: diagnosis, care planning, intervention and evaluation of treatment results.

#### Step 3: Development of process maps

A more detailed process map was developed for each step in the care delivery value chain, mapping processes, activities and measurements throughout the care cycle, including personnel used in each step.

#### Step 4: Process time estimation

The duration for each activity was estimated through time studies (observations and interviews) for each staff category (i.e. coordinating nurse, medical secretaries, psychologists and medical doctors). Time estimates were therefore obtained for each step along the clinical pathway. The results of the time studies yielded a set of standard times for each resource. In addition, the treatment software logged the amount of time that therapists spent working online with each patient, providing access to accurate information regarding resource use during the intervention. Resource use and process times could therefore be calculated from both logged therapist time and time estimates of standardized clinical and administrative tasks, allowing estimation of the variability of total resource use.

#### Step 5: Estimation of resource costs

The costs of resources used in each process were estimated. Data were collected from several sources, including administrative and medical staff, the payroll system, local business intelligence systems and the treatment software database, using 2013 as a reference year for salaries and overhead costs.

#### Step 6: Estimation of resource capacity and capacity cost rates

The practical capacity of each resource was determined. The practical capacity of a resource refers to the amount of time clinically available for the resource (i.e., excluding non-treatment activities such as breaks, meetings and training), as opposed to the theoretical capacity, which is normally 40 hours per week for a full time employee. Practical capacity was estimated to be 80% of the actual number of worked hours per employee, which is typically used as a standard assumption.[13] Calculation of practical capacity was based on attendance and wages per employee, as indicated by the payroll system. Overhead costs of hospital space, supervision, IT and management were allocated over each staff category evenly per minute. In addition, software costs for the treatment platform were allocated only to therapists. Costs for hospital space were calculated as the square meter price divided by floor space per staff category. Joint surfaces were then allocated evenly. Shared costs included management and leadership as well as shared unit administration. Hospital costs for security and safety were also included in the capacity cost rates. Capacity cost rates were operationalized as the minute cost of each resource used [13].

#### Step 7: Calculating the total cost of patient care

The total cost of treating depression with ICBT was calculated as the sum of the total costs of resources used in each process. Costs were calculated in local currency units (SEK) and converted into international dollars[22].

### Effectiveness measurement

Treatment effectiveness was measured using the nine-item Patient Health Questionnaire depression scale (PHQ-9).[23] The PHQ-9 measures nine DSM-IV criteria of depressive disorder on a scale of “0” (not at all) to “3” (nearly every day). Remission was operationalized using established cut-off scores of the PHQ-9 where full remission was defined as a PHQ-9 score of < 5. Sustainability of treatment effect (full remission from depression) was evaluated at 6-month follow-up. Patient satisfaction was measured after treatment with the Client Satisfaction Questionnaire CSQ-8 [24, 25].

### Cost-effectiveness analysis

Cost-effectiveness analysis (CEA) were performed from a healthcare system perspective. The incremental cost-effectiveness ratio (ICER) was estimated at post-treatment and at follow-up. To analyze these ICERs, confidence ellipses at 50%, 75%, and 95% were developed and cost-effectiveness acceptability curves (CEACs) were constructed to represent the uncertainty around estimates [26] in accordance with recommended guidelines [27]. The central limit theorem (CLT) was used in the estimation of the measures within the CEA [18].

The concept of incremental net benefit (INB) was used to interpret the CEAC, where the slope of the net monetary benefits (NMB) curve represents the difference in effects (ΔE) between the two study samples. An intervention was considered as cost-effective only if INB(*K*) > 0, where *K* represents the willingness to pay (WTP) for an additional unit of health gain (one more remission). Since the threshold value of the maximum WTP may be unknown, the INB of the organizational intervention compared to the 2013 processes was plotted and reported with a 95% confidence interval (CI).

### Ethics statement

The study, including its consent procedure (i.e. passive consent), was approved by the Regional Ethical Review Board in Stockholm, Sweden (no 2011/2091-31/3). The ethics committee waived the requirement for active informed consent since this research was conducted as a retrospective cohort study of patients in routine clinical practice. Instead, all participants were informed in writing of the study and given a choice of participation through an opt-out methodology. In addition, patients’ data were anonymized before access by the researchers. Since passive consent does not violate the option of providing choice and increases the likelihood of having a representative sample, this approach is considered to be an efficient procedure for registry data [28, 29].

## Results

### Effectiveness

The number of observations and PHQ-9 means for patients treated during 2013 and 2014 respectively are presented in Table 1.

**Table 1:** Patient characteristics.

	Year	N	Mean	Std. Deviation	Std. Error Mean
**Age (yrs)**	2013	279	37,35	12,32	0,74
	2014	385	37,42	12,08	0,62
**Gender (% males)**	2013	279	30%	0,46	0,03
	2014	385	32%	0,47	0,02
**Minutes spent online per therapist**	2013	280	192,54	111,69	6,67
	2014	386	173,43	126,39	6,43
**Adherence (modules per patient)**	2013	281	7,27	3,06	0,18
	2014	386	7,28	3,03	0,15
**PHQ-9 pre-treatment**	2013	278	14,69	4,97	0,30
	2014	378	14,11	4,88	0,25
**PHQ-9 post-treatment**	2013	243	8,65	5,54	0,36
	2014	325	8,45	5,70	0,32
**PHQ-9 6-month follow-up**	2013	123	6,93	5,88	0,53
	2014	135	6,90	6,03	0,52
**CSQ-8 score**	2013	240	25,05	4,52	0,29
	2014	322	25,43	4,58	0,26

During 2013, PHQ-9 scores decreased by 6.08 (95% CI: 5.38–6.77) between pre- (*M* = 14.69, *SD* = 5.00) and post-treatment assessments (*M* = 8.61, *SD* = 5.52), *p* < .001, corresponding to a within-group effect size of *d* = 1.15 (95% CI: 0.98–1.32). For 2014, PHQ-9 scores decreased by 5.55 (95% CI: 4.94–6.17) between pre- (*M* = 14.00, *SD* = 4.79) and post-treatment assessments (*M* = 8.45, *SD* = 5.70), *p* < .001, corresponding to a within-group effect size of *d* = 1.05 (95% CI: 0.90–1.19). Of patients who provided post-treatment data on the PHQ-9, 27% had achieved full remission from depression after treatment and 42% at follow-up(Table 2); there was no significant difference in the rate of remitters between 2013 and 2014 (*p* > .9). In addition, an additional 35% and 33% had achieved partial remission in 2013 and 2014, respectively. At follow-up, 42% were in full remission during 2013 and 2014; 35% and 33% were in partial remission during 2013 and 2014, respectively. Patient satisfaction (CSQ-8) varied non-significantly between 78% (*M* = 25.05, *SD* = 4.52) in 2013 and 79% (*M* = 25.43, *SD* = 4.58) in 2014 (*p* > .05).

**Table 2:** Calculation of statistics.

	Post-treatment (n = 568)			Follow-up (n = 258)		
	Intervention group (2014)	Control group (2013)	Difference	Intervention group (2014)	Control group (2013)	Difference
**Sample size**	325	243		135	123	
**Effect (full remission)**						
Mean (*SD*)	27.4% (44.7%)	27.2% (44.6%)	0.2%	42.2% (49.6%)	42.3% (49.6%)	-0.1%
Standard error of mean	0.025	0.029	0.038	0.043	0.045	0.062
**Cost ($)**						
Mean (*SD*)	646 (161)	680 (135)	-35	659 (134)	709 (130)	-49
Standard error of mean	9	9	12	12	12	16
**Cost and effect**						
Covariance	-0.191	3.242	0.013	-5.060	-4.028	-0.070
Correlation	-0.003	0.041	0.027	-0.076	-0.062	-0.069
**ICER**			-$15,398			$91,113

Note. *SD*, standard deviation; ICER, Incremental cost-effectiveness ratio.

### Costs

Mean total healthcare costs of ICBT treatment for depression is reported in Table 2. Taking into account the complete healthcare process between referral to follow-up, the estimated average total cost of healthcare decreased from $709 (*SD* = $130) before staff reallocation in 2013 to $659 (*SD* = $134) in 2014. Estimated capacity cost rates (minute prices for each staff category) are presented in Table 3. The two major cost drivers were intervention costs, which contributed up to 54% of the overall costs, and assessment costs, which contributed up to 34% of the overall costs.

**Table 3:** Capacity cost rates for internet-based cognitive therapy in the treatment of depression.

	Coordinating nurse	Medical secretary	Psychologist	Resident physician	Medical specialist
**Capacity cost rate ($/minute)**	$1.25	$1.05	$1.28	$1.44	$2.20

### Cost-effectiveness

**Fig 1:**
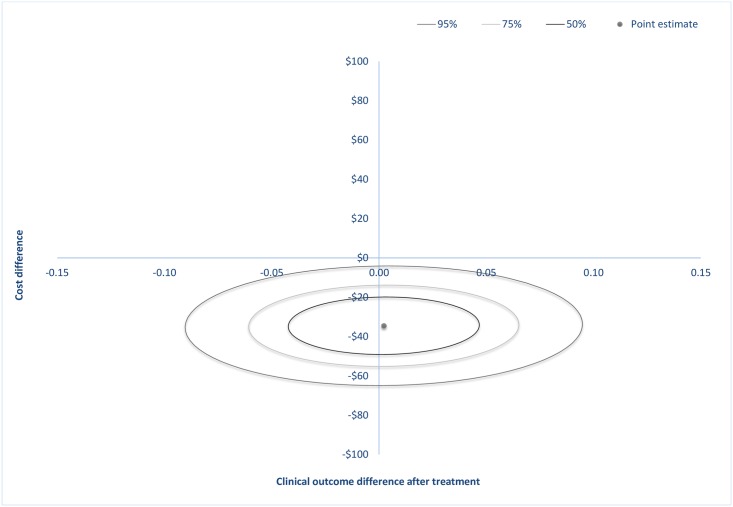
Incremental cost-effectiveness plane showing the mean differences in costs and in the primary outcome measure at post-treatment with 50%, 75% or 95% confidence-ellipses around the point estimate.

**Fig 2:**
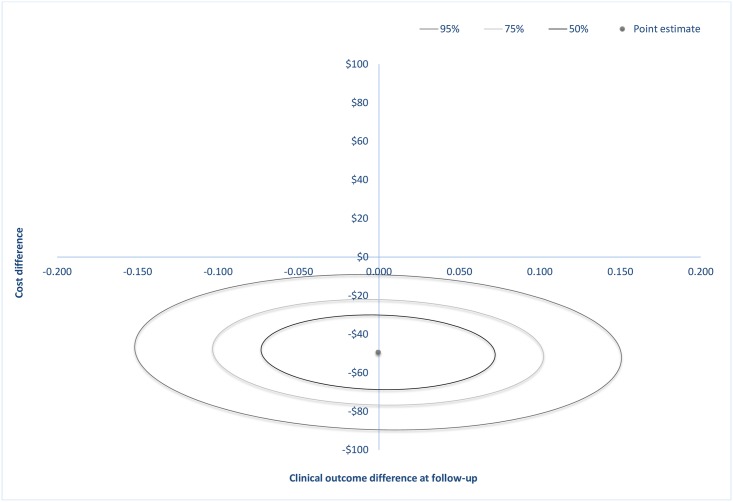
Incremental cost-effectiveness plane showing the mean differences in costs and in the primary outcome measure at follow-up with 50%, 75% or 95% confidence-ellipses around the point estimate.

At post-treatment, the estimated cost-saving was $35 (*SE* = 12) and at follow-up $49 (*SE* = 16). Confidence-ellipses around the point estimate are showed in Fig 1 and in Fig 2 where each ellipse represents regions with a 50%, 75% or 95% probability of containing the true difference in cost and effect. These ellipses occupy two quadrants on the incremental CE plane: the south-east (SE) and south-west (SW) quadrants at both post-treatment and at follow-up, indicating that the ICBT treatment was less costly and equally effective after staff reallocation. The entire density within the ellipses involves cost-savings.

**Fig 3:**
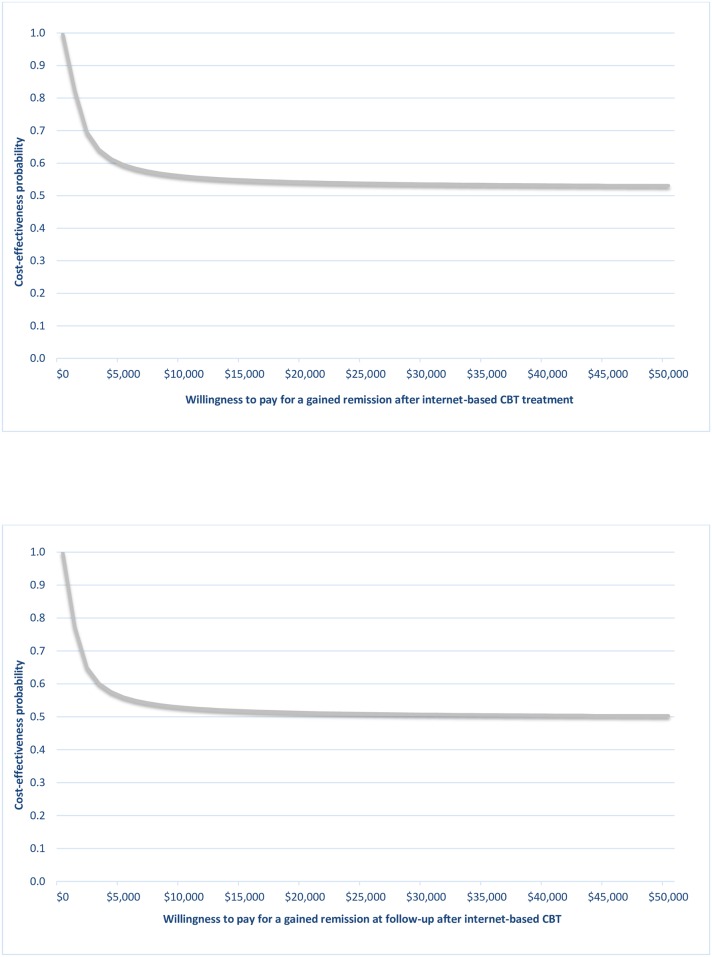
Cost-effectiveness acceptability curves at after treatment and at follow-up based on the willingness to pay for an additional remission from depression achieved, considering the total costs of healthcare.

The cost-effectiveness acceptability curves (CEAC) for post-treatment and follow-up assessments are presented in Fig 3. These indicate the probability that the staff reallocation is cost-effective compared with before the change for a given value of the maximum willingness to pay (WTP) for a gained full remission. It may be observed that the CEACs asymptote to a value less than 1; since only 50% of the joint density involves health gains, the CEAC is decreasing as the WTP increases. This is the case when there is any evidence that the process change could be less effective (i.e. where the joint density (Δ*C*, Δ*E*) of incremental costs and incremental effects is located within the western quadrants) [26].

**Fig 4:**
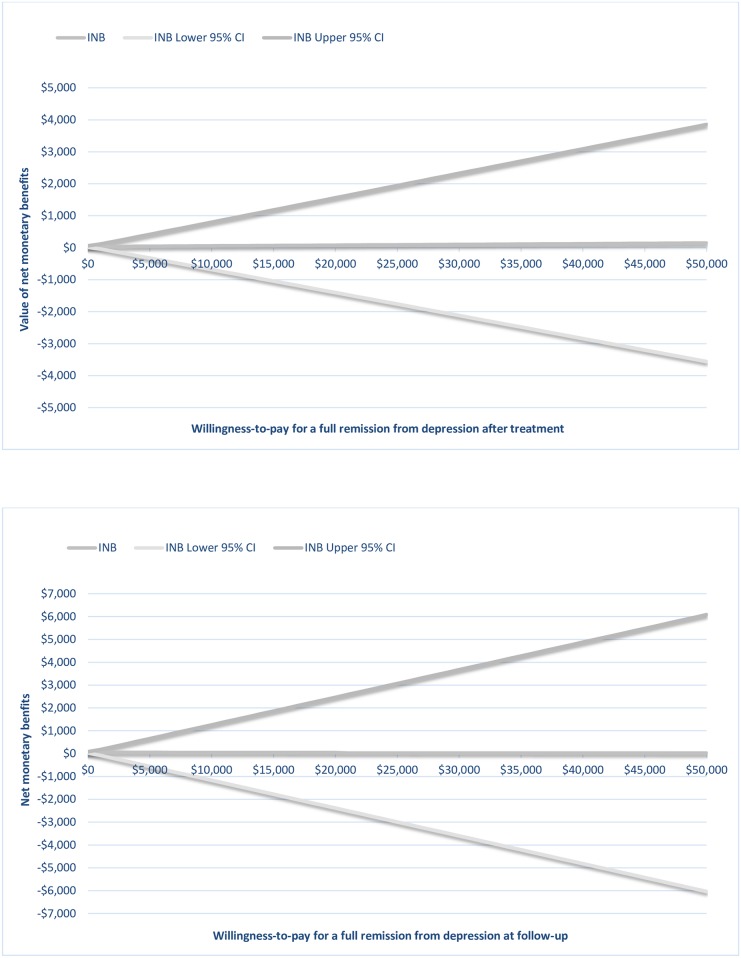
Net monetary benefit curves and 95% confidence intervals at post-treatment and at follow-up.

A graphical representation of the net benefit is illustrated in Fig 4. The uncertainty of the value of the intervention gets larger as the WTP for the clinical outcome increases; this is reflected in the increasing CI of the INB.

The positive NMBs (Table 4) suggest that the intervention is cost-effective, both at post-treatment and at follow-up assessment.

**Table 4:** Incremental net benefit.

	Post-treatment				Follow-up			
WTP	E(INB)	95% CI for INB		Q	E(INB)	95% CI for INB		Q
$0	$35	$10	$59	0.997	$49	$17	$82	0.999
$5 000	$46	-$325	$417	0.595	$47	-$562	$656	0.560
$10 000	$57	-$684	$798	0.560	$44	-$1 170	$1 258	0.528
$15 000	$68	-$1 044	$1 180	0.548	$41	-$1 779	$1 861	0.518
$20 000	$79	-$1 403	$1 562	0.542	$39	-$2 387	$2 464	0.512
$25 000	$91	-$1 763	$1 944	0.538	$36	-$2 995	$3 067	0.509
$30 000	$102	-$2 122	$2 326	0.536	$33	-$3 604	$3 670	0.507
$35 000	$113	-$2 482	$2 708	0.534	$30	-$4 212	$4 273	0.506
$40 000	$124	-$2 841	$3 090	0.533	$28	-$4 821	$4 876	0.504
$45 000	$135	-$3 201	$3 472	0.532	$25	-$5 429	$5 479	0.504
$50 000	$147	-$3 560	$3 854	0.531	$22	-$6 038	$6 082	0.503

Note. WTP, willingness-to-pay; Q, probability of being cost-effective.

## Discussion

This study presents the use of TDABC in CEA to assess the value of process improvement initiatives in mental health care. Previous studies have established the clinical effectiveness of ICBT for depression [20, 30, 31], and the estimated effect sizes in the present study (*d* = 1.05–1.15) are in line with these. The main objective in this study was to observe whether treatment effectiveness can be sustained as doctors, who are in short supply and high demand, are replaced with psychologists for the activity of post-treatment assessment. Results indicated that healthcare costs decreased from $709 per treated case to $659 while treatment effects were maintained with 47% of patients achieving full remission at follow-up. Confidence ellipses occupied the south-east (SE) and south-west (SW) quadrants of the incremental cost-effectiveness plane at both post-treatment and at follow-up, indicating cost-saving where providing healthcare became less costly but equally effective after staff reallocation. The absence of an observed increase in effectiveness is reflected in the flatness of the slope of the INB line (Fig 4). Since the NMB values are positive, the process improvement initiative may be considered as cost-effective; however, the widening CI for the NMB line indicate an increasing uncertainty around the value of the intervention as the WTP threshold gets larger. In other words, the more value decision makers place on achieving better health outcomes, the less we can say about the value of the process change. However, as discussed above, the purpose of this intervention was not to increase effectiveness of the intervention, but rather to maintain the same level of effectiveness as before. In addition, it was observed that patient waiting times were reduced by approximately one week to be scheduled for a diagnostic interview and to start treatment; the median time that a patient waited for a diagnostic assessment decreased from 22 days in 2013 to 16 days in 2014 and RTT decreased from 28 days in 2013 to 23 days in 2014. Finally, patient satisfaction (from 78% to 79%) remained constant after staff reallocation.

Although these results complement previous research demonstrating that ICBT is associated with improved clinical and economic outcomes [3], most economic evaluations of the treatment of depression have been performed from a societal perspective, without a detailed analysis of the actual resources used throughout the care delivery cycle. Increased accuracy of cost assessments in mental health care is important in understanding the actual costs of care because such information could yield valuable insights in identifying the resources driving these costs. The purpose of using TDABC, therefore, was to achieve a more accurate method of estimating resource utilization and costs of healthcare delivery compared to more traditional costing methods such as ABC, which does not take into account the time required to perform activities in healthcare processes. By using TDABC in conjunction with established methods of CEA, we assessed its usability as a viable method for quantifying the value achieved in value-based healthcare [7] and taking into account the uncertainty surrounding estimated benefits of process improvement initiatives. Additional benefits of using the TDABC approach may be that healthcare providers gain a better understanding of how activities and resources are accumulated into patient level costs. Detailed information on the costs of healthcare processes may benefit strategic decision-making, allowing more insight into which process improvement initiatives are likely to lead to maintaining or improving health outcomes given available or less costly resources. For example, through this study, managers and staff at ICBT unit was provided with a detailed description of the unit’s care delivery process, its constituent parts, the resources used, their costs and the clinical outcomes. The methodology may therefore offer providers with an overall understanding of the components of their delivery system, enabling comparison and evaluation of the relative importance of each component; after all, efforts to increase efficiency in mental healthcare involves not merely cost-cutting strategies but rather improving resource allocation and utilization. Incorporating TDABC in CEA may offer further insights into which processes or resources may potentially contribute the most in terms of patient value.

We hope that this study may contribute to an understanding of—or stimulate further discussion on—how to best apply and measure the concept of “value” in mental healthcare by integrating established CEA methods into the value-based healthcare framework. The visualization of value, either on a CE plane as the widely adopted standard for health economic evaluation [16, 17] or on a radar chart, as proposed by Kaplan and colleagues [15], provides a fast and intuitive snapshot on the relative costs and effects between alternatives. As estimated costs and effects always involves uncertainty, we believe that presenting or reporting CIs should be included as these provide another important dimension as to the meaning of the estimates. Finally, we believe that standardized methods and guidelines of how to measure and reporting value within healthcare should be developed and agreed upon within the research community as to facilitate comparison across healthcare providers or across studies of value-based healthcare.

This study has some limitations. First, although TDABC has been extensively applied to industry [7, 9, 10], it is relatively new in healthcare, particularly mental health care. This study focused on one clinical unit, and time and cost estimates might differ significantly from other organizations within the mental healthcare field because processes differ and are not consistently standardized across healthcare providers. As a consequence, it may be difficult to generalize estimated costs of treatment or of the total healthcare process. Nevertheless, the results of this study may serve as a benchmark for other healthcare providers. In addition, one advantage of the TDABC method of measuring treatment costs—compared with traditional activity-based costing (ABC) [32]–is that by inventorying each step of the care process, inefficient processes within the care delivery cycle may be more easily identified. Another limitation concerns the difficulties to accurately assess time and costs involved in healthcare processes. Although accurate timing of the amount of time each psychologist spent with each patient, the time estimates and costing of other administrative processes were based on averages.

To conclude, this study illustrates how a value-based framework can be implemented in a mental healthcare setting by using TDABC as a potentially more accurate costing method in conjunction with established CEA methodology. The results also showed that the quality of care could be maintained after having replaced scarce psychiatrists with more available psychologists. We encourage further studies of the use of these methods within mental health care.

## Supporting Information

S1 Dataset Combined data set at post-treatment. (PDF)

S2 Dataset Combined data set at follow-up. (PDF)
