# Supplementary material for: Combining Time-Driven Activity-Based Costing with Clinical Outcome in Cost-Effectiveness Analysis to Measure Value in Treatment of Depression
Source: PLoS One. 2016 Oct 31;11(10):e0165389. doi: 10.1371/journal.pone.0165389 (PMC5087942; doi:10.1371/journal.pone.0165389)
Supplement: S1 Dataset — (PDF) [file pone.0165389.s001.pdf]

Control Group (2013)				Intervention Group (2014)		
**Patient**	**Effect (achieved full remission [PHQ-9<5] at post-treatment)**	**Cost ($)**		**Patient**	**Effect (achieved full remission [PHQ-9<5] at post-treatment)**	**Cost ($)**
**1**	0	708,76		1	0	734,41
**2**	0	814,25		2	0	1342,48
**3**	0	613,10		3	0	782,52
**4**	0	703,24		4	0	617,32
**5**	0	669,00		5	0	657,49
**6**	0	754,52		6	0	569,29
**7**	0	909,57		7	0	708,28
**8**	0	636,72		8	0	576,23
**9**	0	560,62		9	0	594,19
**10**	0	789,36		10	0	963,62
**11**	0	625,24		11	0	610,83
**12**	0	799,58		12	0	570,02
**13**	0	713,88		13	0	581,33
**14**	0	733,72		14	0	688,89
**15**	0	769,50		15	0	437,73
**16**	0	832,22		16	0	682,30
**17**	0	826,56		17	0	551,59
**18**	0	499,35		18	0	685,22
**19**	0	641,05		19	0	651,53
**20**	0	647,19		20	0	672,21
**21**	0	899,87		21	0	603,81
**22**	0	890,46		22	0	677,75
**23**	0	781,04		23	0	822,59
**24**	0	757,29		24	0	676,49
**25**	0	677,49		25	0	801,96
**26**	0	683,52		26	0	795,02
**27**	0	598,25		27	0	506,66
**28**	0	587,63		28	0	629,39
**29**	0	482,12		29	0	480,12
**30**	0	772,12		30	0	1136,51
**31**	0	700,36		31	0	588,92
**32**	0	633,95		32	0	531,47
**33**	0	571,76		33	0	620,67
**34**	0	824,79		34	0	730,55
**35**	0	507,14		35	0	476,75
**36**	0	847,53		36	0	732,92
**37**	0	558,70		37	0	802,49
**38**	0	859,05		38	0	610,87
**39**	0	528,75		39	0	567,37
**40**	0	981,83		40	0	620,47
**41**	0	630,06		41	0	630,78
**42**	0	541,10		42	0	686,22
**43**	0	591,83		43	0	703,18
**44**	0	939,40		44	0	752,44
**45**	0	912,13		45	0	559,87
**46**	0	550,43		46	0	637,31

**47**	0	612,53		47	0	562,94
**48**	0	601,60		48	0	677,58
**49**	0	740,53		49	0	753,59
**50**	0	751,75		50	0	666,36
**51**	0	801,65		51	0	609,96
**52**	0	725,74		52	0	727,10
**53**	0	884,78		53	0	598,33
**54**	0	662,70		54	0	701,20
**55**	0	673,88		55	0	464,48
**56**	0	699,69		56	0	482,72
**57**	0	818,82		57	0	521,08
**58**	0	630,89		58	0	617,72
**59**	0	701,53		59	0	813,99
**60**	0	742,51		60	0	894,80
**61**	0	747,27		61	0	477,80
**62**	0	608,03		62	0	852,54
**63**	0	519,81		63	0	677,95
**64**	0	709,06		64	0	902,50
**65**	0	752,62		65	0	950,76
**66**	0	616,52		66	0	636,52
**67**	0	694,11		67	0	680,14
**68**	0	526,23		68	0	604,49
**69**	0	724,23		69	0	871,14
**70**	0	682,80		70	0	565,82
**71**	0	616,92		71	0	610,04
**72**	0	684,36		72	0	612,41
**73**	0	653,40		73	0	904,80
**74**	0	522,61		74	0	606,05
**75**	0	654,85		75	0	657,96
**76**	0	736,69		76	0	660,81
**77**	0	449,69		77	0	628,03
**78**	0	723,95		78	0	570,30
**79**	0	640,22		79	0	564,52
**80**	0	766,53		80	0	746,34
**81**	0	757,64		81	0	925,20
**82**	0	676,38		82	0	650,11
**83**	0	671,13		83	0	586,06
**84**	0	859,42		84	0	633,19
**85**	0	471,09		85	0	646,35
**86**	0	610,67		86	0	738,28
**87**	0	612,80		87	0	867,83
**88**	0	900,10		88	0	516,28
**89**	0	427,31		89	0	617,98
**90**	0	447,58		90	0	572,52
**91**	0			91	0	521,47
**92**	0	1053,76		92	0	592,63
**93**	0	605,76		93	0	562,36
**94**	0	647,64		94	0	559,91
**95**	0	654,92		95	0	623,31
**96**	0	547,48		96	0	701,07

**97**	0	796,68		97	0	568,93
**98**	0	496,81		98	0	606,91
**99**	0	635,91		99	0	990,80
**100**	0	655,41		100	0	530,55
**101**	0	818,61		101	0	596,37
**102**	0	622,49		102	0	644,07
**103**	0	759,62		103	0	563,36
**104**	0	602,22		104	0	597,75
**105**	0	803,50		105	0	728,04
**106**	0	517,02		106	0	565,13
**107**	0	469,85		107	0	506,92
**108**	0	541,10		108	0	566,61
**109**	0	500,65		109	0	400,04
**110**	0	928,64		110	0	789,16
**111**	0	852,36		111	0	739,09
**112**	0	564,42		112	0	558,39
**113**	0	580,14		113	0	574,91
**114**	0	684,89		114	0	619,17
**115**	0	768,96		115	0	695,31
**116**	0	626,69		116	0	697,72
**117**	0	585,26		117	0	501,18
**118**	0	665,73		118	0	607,95
**119**	0	702,06		119	0	746,60
**120**	0	731,46		120	0	738,51
**121**	0	466,29		121	0	511,78
**122**	0	627,14		122	0	533,31
**123**	0	559,51		123	0	932,32
**124**	0	503,83		124	0	992,76
**125**	0	559,04		125	0	575,80
**126**	0	504,77		126	0	708,32
**127**	0	653,44		127	0	737,17
**128**	0	678,25		128	0	640,70
**129**	0	481,86		129	0	518,91
**130**	0	574,38		130	0	680,01
**131**	0	537,37		131	0	514,60
**132**	0	788,21		132	0	644,49
**133**	0	659,1		133	0	553,81
**134**	0	772,36		134	0	413,41
**135**	0	931,99		135	0	550,73
**136**	0	671,04		136	0	788,37
**137**	0	888,17		137	0	505,17
**138**	0	579,89		138	0	472,21
**139**	0	537,58		139	0	565,63
**140**	0	898,91		140	0	556,49
**141**	0	500,97		141	0	954,11
**142**	0	518,19		142	0	577,76
**143**	0	865,13		143	0	971,17
**144**	0	509,61		144	0	528,76
**145**	0	558,34		145	0	498,53
**146**	0	958,85		146	0	830,39

**147**	0	969,2		147	0	685,99
**148**	0	857,63		148	0	575,48
**149**	0	619,16		149	0	713,32
**150**	0	995,18		150	0	622,76
**151**	0	804,51		151	0	427,58
**152**	0	561,43		152	0	449,38
**153**	0	749,06		153	0	635,43
**154**	0	662,17		154	0	641,59
**155**	0	596,95		155	0	581,01
**156**	0	599,36		156	0	535,89
**157**	0	688,56		157	0	604,49
**158**	0	622,98		158	0	664,40
**159**	0	680,32		159	0	577,06
**160**	0	633,99		160	0	528,61
**161**	0	626,76		161	0	653,13
**162**	0	604,61		162	0	492,07
**163**	0	754,14		163	0	574,88
**164**	0	649,18		164	0	606,29
**165**	0	668,7		165	0	652,71
**166**	0	585,88		166	0	477,50
**167**	0	562,48		167	0	489,02
**168**	0	828,25		168	0	560,44
**169**	0	759,02		169	0	575,42
**170**	0	580,4		170	0	552,53
**171**	0	622,81		171	0	578,53
**172**	0	688,37		172	0	711,48
**173**	0	547,25		173	0	621,84
**174**	0	458,82		174	0	550,76
**175**	0	627,42		175	0	632,93
**176**	0	682,88		176	0	613,24
**177**	0	531,48		177	0	768,42
**178**	1	601,56		178	0	547,43
**179**	1	694,02		179	0	659,17
**180**	1	586,22		180	0	599,20
**181**	1	747,67		181	0	595,73
**182**	1	892,25		182	0	533,50
**183**	1	810,93		183	0	603,07
**184**	1	715,35		184	0	540,75
**185**	1	568,77		185	0	577,42
**186**	1	667,2		186	0	750,12
**187**	1	730,22		187	0	611,36
**188**	1	762,8		188	0	556,13
**189**	1	579,25		189	0	599,84
**190**	1	830,3		190	0	657,55
**191**	1	726,06		191	0	519,72
**192**	1	832,71		192	0	639,99
**193**	1	699,25		193	0	523,17
**194**	1	1193,31		194	0	945,38
**195**	1	1141,34		195	0	450,57
**196**	1	644,78		196	0	624,76

**197**	1	801,24		197	0	561,98
**198**	1	739,65		198	0	983,31
**199**	1	740,36		199	0	558,16
**200**	1	518,55		200	0	713,64
**201**	1	865,45		201	0	666,02
**202**	1	670,89		202	0	532,11
**203**	1	579,63		203	0	885,99
**204**	1	739,37		204	0	588,13
**205**	1	722,93		205	0	779,77
**206**	1	543,17		206	0	475,32
**207**	1	801,92		207	0	407,42
**208**	1	843,44		208	0	503,44
**209**	1	889,48		209	0	620,62
**210**	1	641,31		210	0	638,14
**211**	1	783,64		211	0	511,52
**212**	1	625,92		212	0	548,22
**213**	1	490,39		213	0	461,01
**214**	1	909,57		214	0	764,73
**215**	1	732,74		215	0	473,96
**216**	1	729,03		216	0	742,03
**217**	1	520,43		217	0	742,39
**218**	1	567,21		218	0	464,51
**219**	1	882,27		219	0	722,53
**220**	1	535,83		220	0	921,57
**221**	1	843,29		221	0	891,09
**222**	1	505,82		222	0	866,87
**223**	1	613,55		223	0	490,19
**224**	1	569,56		224	0	531,53
**225**	1	558,15		225	0	847,44
**226**	1	551,83		226	0	601,42
**227**	1	663,24		227	0	489,21
**228**	1	614,68		228	0	1511,03
**229**	1	658,48		229	0	434,64
**230**	1	549,25		230	0	538,32
**231**	1	512,32		231	0	445,58
**232**	1	1014,92		232	0	545,12
**233**	1	530,56		233	0	533,67
**234**	1	639,6		234	0	674,89
**235**	1	510,96		235	0	688,59
**236**	1	468,06		236	0	1030,37
**237**	1	560,6		237	1	905,74
**238**	1	652,1		238	1	581,45
**239**	1	588,85		239	1	679,84
**240**	1	724,25		240	1	607,93
**241**	1	553,05		241	1	753,53
**242**	1	700,14		242	1	525,45
**243**	1	602,63		243	1	531,94
				244	1	571,68
				245	1	621,05
				246	1	682,55

				247	1	705,21
				248	1	555,75
				249	1	590,37
				250	1	588,67
				251	1	616,87
				252	1	589,18
				253	1	606,78
				254	1	639,87
				255	1	529,21
				256	1	715,85
				257	1	619,92
				258	1	606,95
				259	1	695,55
				260	1	562,53
				261	1	771,21
				262	1	697,15
				263	1	579,77
				264	1	928,95
				265	1	602,53
				266	1	680,25
				267	1	541,95
				268	1	658,30
				269	1	606,37
				270	1	596,03
				271	1	407,52
				272	1	704,74
				273	1	567,84
				274	1	463,08
				275	1	535,48
				276	1	643,09
				277	1	638,39
				278	1	396,90
				279	1	468,47
				280	1	593,79
				281	1	673,74
				282	1	558,82
				283	1	520,80
				284	1	509,16
				285	1	751,55
				286	1	634,32
				287	1	537,74
				288	1	540,05
				289	1	819,04
				290	1	499,62
				291	1	772,41
				292	1	811,39
				293	1	651,07
				294	1	637,48
				295	1	596,49
				296	1	628,37

				297	1	531,17
				298	1	581,05
				299	1	584,10
				300	1	635,47
				301	1	619,81
				302	1	597,82
				303	1	613,48
				304	1	633,10
				305	1	532,22
				306	1	591,35
				307	1	710,69
				308	1	442,55
				309	1	700,09
				310	1	430,41
				311	1	477,31
				312	1	461,97
				313	1	819,98
				314	1	712,74
				315	1	444,86
				316	1	575,78
				317	1	1271,91
				318	1	1731,32
				319	1	603,28
				320	1	672,87
				321	1	458,17
				322	1	806,67
				323	1	1256,83
				324	1	718,29
				325	1	869,43
	**Mean**	**Mean**			**Mean**	**Mean**
	**,27**	**680,07**			**,27**	**645,56**
