# Supplementary material for: Combining Time-Driven Activity-Based Costing with Clinical Outcome in Cost-Effectiveness Analysis to Measure Value in Treatment of Depression
Source: PLoS One. 2016 Oct 31;11(10):e0165389. doi: 10.1371/journal.pone.0165389 (PMC5087942; doi:10.1371/journal.pone.0165389)
Supplement: S2 Dataset — (PDF) [file pone.0165389.s002.pdf]

Control Group (2013)				Intervention Group (2014)		
**Patient**	**Effect (achieved full remission [PHQ-9<5] at follow-up)**	**Cost ($)**		**Patient**	**Effect achieved full remission [PHQ-9<5] at follow-up)**	**Cost ($)**
**1**	0	708,76		1	0	734,41
**2**	0	814,25		2	0	1342,48
**3**	0	703,24		3	0	657,49
**4**	0	754,52		4	0	569,29
**5**	0	909,57		5	0	905,74
**6**	0	560,62		6	0	594,19
**7**	0	789,36		7	0	963,62
**8**	0	625,24		8	0	688,89
**9**	0	733,72		9	0	437,73
**10**	0	769,50		10	0	607,93
**11**	0	892,25		11	0	551,59
**12**	0	832,22		12	0	603,81
**13**	0	810,93		13	0	676,49
**14**	0	826,56		14	0	801,96
**15**	0	499,35		15	0	795,02
**16**	0	641,05		16	0	506,66
**17**	0	667,20		17	0	629,39
**18**	0	899,87		18	0	682,55
**19**	0	890,46		19	0	555,75
**20**	0	781,04		20	0	480,12
**21**	0	757,29		21	0	1136,51
**22**	0	677,49		22	0	531,47
**23**	0	598,25		23	0	476,75
**24**	0	587,63		24	0	732,92
**25**	0	772,12		25	0	802,49
**26**	0	700,36		26	0	610,87
**27**	0	633,95		27	0	620,47
**28**	0	507,14		28	0	616,87
**29**	0	726,06		29	0	589,18
**30**	0	859,05		30	0	630,78
**31**	0	1193,31		31	0	686,22
**32**	0	1141,34		32	0	703,18
**33**	0	528,75		33	0	639,87
**34**	0	981,83		34	0	752,44
**35**	0	630,06		35	0	559,87
**36**	0	541,10		36	0	637,31
**37**	0	591,83		37	0	562,94
**38**	0	939,40		38	0	562,53
**39**	0	912,13		39	0	753,59
**40**	0	612,53		40	0	666,36
**41**	0	601,60		41	0	727,10
**42**	0	740,53		42	0	598,33
**43**	0	751,75		43	0	697,15
**44**	0	801,65		44	0	701,20
**45**	0	725,74		45	0	521,08
**46**	0	662,70		46	0	813,99

**47**	0	699,69		47	0	477,80
**48**	0	747,27		48	0	852,54
**49**	0	608,03		49	0	677,95
**50**	0	519,81		50	0	680,25
**51**	0	752,62		51	0	902,50
**52**	0	616,52		52	0	636,52
**53**	0	694,11		53	0	541,95
**54**	0	526,23		54	0	604,49
**55**	0	724,23		55	0	871,14
**56**	0	682,80		56	0	610,04
**57**	0	684,36		57	0	612,41
**58**	0	653,40		58	0	904,80
**59**	0	522,61		59	0	606,05
**60**	0	843,44		60	0	657,96
**61**	0	654,85		61	0	660,81
**62**	0	736,69		62	0	628,03
**63**	0	900,10		63	0	606,37
**64**	0	647,64		64	0	570,30
**65**	0	626,69		65	0	564,52
**66**	0	671,04		66	0	407,52
**67**	0	561,43		67	0	650,11
**68**	0	599,36		68	0	567,84
**69**	0	626,76		69	0	586,06
**70**	0	649,18		70	0	597,75
**71**	0	585,88		71	0	932,32
**72**	1	613,10		72	0	641,59
**73**	1	669,00		73	0	581,01
**74**	1	636,72		74	0	604,49
**75**	1	601,56		75	0	653,13
**76**	1	799,58		76	0	619,81
**77**	1	713,88		77	0	750,12
**78**	1	694,02		78	0	742,03
**79**	1	586,22		79	1	782,52
**80**	1	747,67		80	1	617,32
**81**	1	715,35		81	1	708,28
**82**	1	568,77		82	1	576,23
**83**	1	647,19		83	1	610,83
**84**	1	730,22		84	1	570,02
**85**	1	683,52		85	1	581,45
**86**	1	762,80		86	1	581,33
**87**	1	482,12		87	1	679,84
**88**	1	579,25		88	1	682,30
**89**	1	733,91		89	1	685,22
**90**	1	830,30		90	1	753,53
**91**	1	571,76		91	1	651,53
**92**	1	824,79		92	1	672,21
**93**	1	847,53		93	1	525,45
**94**	1	832,71		94	1	677,75
**95**	1	558,70		95	1	822,59
**96**	1	699,25		96	1	531,94

**97**	1	644,78		97	1	571,68
**98**	1	801,24		98	1	621,05
**99**	1	550,43		99	1	705,21
**100**	1	739,65		100	1	588,92
**101**	1	884,78		101	1	620,67
**102**	1	740,36		102	1	479,33
**103**	1	673,88		103	1	730,55
**104**	1	818,82		104	1	590,37
**105**	1	630,89		105	1	588,67
**106**	1	701,53		106	1	567,37
**107**	1	742,51		107	1	606,78
**108**	1	518,55		108	1	529,21
**109**	1	865,45		109	1	715,85
**110**	1	709,06		110	1	619,92
**111**	1	670,89		111	1	606,95
**112**	1	579,63		112	1	695,55
**113**	1	739,37		113	1	677,58
**114**	1	722,93		114	1	771,21
**115**	1	543,17		115	1	609,96
**116**	1	616,92		116	1	579,77
**117**	1	801,92		117	1	464,48
**118**	1	889,48		118	1	482,72
**119**	1	641,31		119	1	928,95
**120**	1	882,27		120	1	602,53
**121**	1	995,18		121	1	617,72
**122**	1	409,86		122	1	894,80
**123**	1	724,25		123	1	950,76
				124	1	680,14
				125	1	658,30
				126	1	565,82
				127	1	746,34
				128	1	596,03
				129	1	925,20
				130	1	704,74
				131	1	701,07
				132	1	664,40
				133	1	633,10
				134	1	430,41
				135	1	477,31
	**Mean**	**Mean**			**Mean**	**Mean**
	**,42**	**708,85**			**,42**	**659,47**
